# A two-sample Mendelian randomization study reveals the causal effects of statin medication on gut microbiota abundance in the European population

**DOI:** 10.3389/fgene.2024.1380830

**Published:** 2024-12-13

**Authors:** Peng Zhou, Chen Qiu, Zequn Zhuang, Kaihang Shi, Zhihui Yang, Yuyan Ding, Huiheng Qu, Jiazeng Xia

**Affiliations:** ^1^ Department of General Surgery, The Affiliated Wuxi No.2 People's Hospital of Nanjing Medical University, Wuxi Medical Center, Nanjing Medical University, Wuxi, Jiangsu, China; ^2^ Department of General Surgery, Institute of General Surgical Research, Jiangnan University Medical Center, School of Medicine, Jiangnan University, Wuxi, China; ^3^ Department of Hepatobiliary Surgery, Jinjiang Municipal Hospital (Shanghai Sixth People’s Hospital Fujian), Quanzhou, Fujian, China; ^4^ Department of Hepatobiliary Surgery, The Affiliated Yixing Hospital of Jiangsu University, Wuxi, China

**Keywords:** causal effect, gut microbiota, Mendelian randomization, statin medication, two-sample Mendelian randomization

## Abstract

**Background:**

Observational studies have reported changes in gut microbiota abundance caused by long-term statin medication therapy. However, the causal relation between statin medication and gut microbiota subsets based on genetic variants remains unclear.

**Methods:**

We used genome-wide association study (GWAS) data on statin medication from the FinnGen database and gut microbiota abundance GWAS data from the IEU OpenGWAS project. A Mendelian randomization (MR) analysis was conducted to evaluate the causal effect of statin medication on gut microbiota abundance using the inverse variance weighting (IVW) method, MR-Egger regression, and weighted median approach. Meanwhile, heterogeneity and pleiotropy analyses were also undertaken in this study.

**Results:**

Statin medication was negatively correlated with five species of gut microbiota abundance: *Parabacteroides* (Beta_IVW_ = −0.2745, 95% CI = (−0.4422, −0.1068), and *P*_IVW_ = 0.0013), *Ruminococcaceae UCG-009* (Beta_IVW_ = −0.1904, 95% CI = (−0.3255, −0.0553), and *P*_IVW_ = 0.0057), *Coprococcus 1* (Beta_IVW_ = −0.1212, 95% CI = (−0.2194, −0.0231), and *P*_IVW_ = 0.0154), *Ruminococcaceae UCG-010* (Beta_IVW_ = −0.1149, 95% CI = (−0.2238, −0.0060), and *P*_IVW_ = 0.0385), and *Veillonellaceae* (Beta_IVW_ = −0.0970, 95% CI = (−0.2238, 0.0060), and *P*_IVW_ = 0.0400) and positively correlated with one species of gut microbiota: *Desulfovibrio* (Beta_IVW_ = 0.2452, 95% CI = (0.0299, 0.4606), and *P*_IVW_ = 0.0255). In addition, no significant heterogeneity or pleiotropy was detected in the abovementioned gut microbiota.

**Conclusion:**

This Mendelian randomization analysis indicates a causal relationship between statin medication and six gut microbiota species. These findings may provide new strategies for health monitoring in populations taking long-term statin medications.

## 1 Introduction

The human gut is colonized with a large number of microorganisms and contains a wide variety of microbial communities, including archaea, fungi, protozoa, and viruses, collectively known as the gut microbiota (Costello et al., 2012). In healthy hosts, the gut microbiota can generally be categorized into five groups according to the phylum level: *Bacteroidota*, *Bacillota*, *Fusobacteria*, *Proteobacteria*, and *Actinobacteria* (Irrazábal et al., 2014). The gut microbiota is characterized by symbiosis, stability, diversity, and integrity, with its composition being highly variable among individuals. This variability is influenced by diet, lifestyle, environment, and genetic factors (Adak and Khan, 2019). As the most important bioactive component of the human gastrointestinal microecosystem, the gut microbiota exists in a symbiotic and mutually beneficial relationship with the host, playing a variety of important physiological roles. The gut microbiota can regulate host energy homeostasis by participating in food digestion and nutrient absorption (Fan and Pedersen, 2021). It can also act like an endocrine organ, regulating the body’s immune response and physiological processes by secreting immune factors and neurotransmitters (Nagata et al., 2023). In addition, the gut microbiota can maintain the integrity of the intestinal mucosal barrier and reduce the translocation of metabolic toxic byproducts (Leibovitzh et al., 2022). Alterations in the composition and abundance of gut microbiota can induce chronic inflammatory responses and diseases by disrupting energy homeostasis, increasing the permeability of the intestinal mucosal barrier, or causing immune dysregulation (Cai et al., 2022). A growing body of research has shown that gut microbiota dysbiosis is associated with a wide range of human diseases, including obesity (Gomes et al., 2018), diabetes (Byndloss et al., 2024), cancer (Han et al., 2023), polycystic ovarian syndrome (Gu et al., 2022a; Gu et al., 2022b), cardiovascular disease (Caparrós-Martín et al., 2024; Hu et al., 2024), and acute coronary syndrome (Guan et al., 2024). Therefore, maintaining the balance and restabilizing the gut microbiota is expected to be a novel, potential therapeutic target for disease prevention, intervention, and drug sensitization. In the pharmacological treatment of the disease, the gut microbiota often serves as a communication medium for the interaction between the drug and the organism. On one hand, multiple studies have found that drugs mediate various metabolic processes by regulating the gut microbiota of the host and further modulate the transduction of the downstream signaling pathways, thus playing a role in delaying and improving the disease (Xu et al., 2020; Wu et al., 2020). For example, vitamin D is critically linked to the human microbiome, which influences the immune system primarily through the vitamin D receptor (VDR), and deficiency of vitamin D alters the microbiome composition and intestinal epithelial immune barrier integrity (Murdaca et al., 2021; Murdaca et al., 2024). On the other hand, certain gut microbiota (e.g., *Akkermansia*, *Bifidobacterium*, *Lactobacillus*, and *Parabacteroides*) also act as drug sensitizers, especially in cancer immunotherapy. These microbiotas not only attenuate toxic side effects in immunotherapy but also suggest the potential for the gut microbiota to serve as a prognostic biomarker for tumor immunotherapy (Wu et al., 2022; Derosa et al., 2022). In addition, specific groups of gut microbiota can be further isolated and treated as probiotic drugs, thereby strengthening the mucosal barrier, promoting the host’s innate and acquired immune responses, and modulating cellular dynamics (Sassone-Corsi and Raffatellu, 2015). Thus, there appears to be a mutually beneficial potential relationship between gut microbiota and drugs, which makes it important to consider the potentially reinforcing effects of gut microbiota during drug therapy.

Statin medication is widely recognized as a class of 3-hydroxy-3-methylglutaryl coenzyme A reductase (HMGCR) inhibitors, which is widely used in the treatment of hyperlipidemia and the prevention of cardiovascular and cerebrovascular diseases (Bibbins-Domingo et al., 2016). Statin medication inhibits the production of mevalonic acid, thus reducing intracellular cholesterol synthesis and lowering total cholesterol and low-density lipoprotein levels (Yu and Liao, 2022; Georgescu et al., 2024). In recent years, with the widespread use and the deepening of research on statins, their “versatility” has been gradually explored in the clinical application of a variety of diseases. For example, statins have been found to exert their antioxidant effects through the regulation of intracellular hydrogen peroxide (H_2_O_2_) via NADPH oxidase (Nox) isozymes (Waldeck-Weiermair et al., 2022), which is consistent with clinical findings that statins significantly reduce the risk of cardiovascular accidents and amputations (Lo et al., 2022). In cancer-related studies, statins have been found to inhibit tumor proliferation by antagonizing cholesterol-related signaling pathways (Pourlotfi et al., 2022) and significantly reduce postoperative mortality in malignant tumors (Saito et al., 2023). Notably, numerous studies (Caparrós-Martín et al., 2017; Dias et al., 2020; Wilmanski et al., 2022) have found that statin therapy can cause changes in the gut microbiota; for example, a multi-omics study (Hu et al., 2021) found a decrease in potentially pathogenic bacteria and an increase in beneficial bacteria in patients with therapeutic acute coronary syndromes receiving statin therapy. However, the gut microbiota profiles modulated by statin medication identified so far are not sufficiently diverse, and further identification is needed. Meanwhile, more robust evidence is needed to support the role of statin medication in modulating the abundance of gut microbiota (Vieira-Silva et al., 2020).

Mendelian randomization (MR) is an epidemiological investigation tool that combines the instrumental variables approach with Mendelian laws of inheritance to explore the causal relationship between exposures and outcomes (Zheng et al., 2017). This tool relies on the principle that “alleles are randomly assigned to offspring at the time of conception” (Sekula et al., 2016; Cornish et al., 2019), allowing it to overcome the interference of unquantifiable and unknown confounding factors that may arise during the exploration of disease etiology (Davies et al., 2018). Compared with randomized controlled studies, MR does not require artificial intervention in the populations (analysis using existing genome-wide association study (GWAS) data) and overcomes the constraints of high research costs and ethical reviews (Gala and Tomlinson, 2020). Moreover, MR determines the sequence of exposures and outcomes ahead of the analysis, avoiding causal inversion in etiological inferences. With the progress and popularity of sequencing technologies, genome-wide association studies have been widely used to study genetic susceptibility to critical diseases and phenotypes, e.g., UK Biobank (http://www.nealelab.is/uk-biobank/) and FinnGen (http://www.nealelab.is/uk-biobank/) (Kurki et al., 2023), identifying a large number of genetic variants and advancing the development of MR analysis.

Hence, we conducted multiple two-sample Mendelian randomization analyses to explore the causal relationship between statin medication and gut microbiota abundance in this study and identified a causal relationship between statins and the abundance of eight gut microbiota taxa from the perspective of genetic susceptibility.

## 2 Methods

### 2.1 Genome-wide association study summary datasets

GWAS refers to the identification of sequence variants, such as single-nucleotide polymorphisms (SNPs), present in the whole human genome, from which SNPs associated with diseases are screened. The latest statin medication GWAS dataset (n = 377,277; 127,169 cases; and 250,108 controls) was downloaded from the FinnGen research project (https://www.finngen.fi/fi), and it finitely mentioned that the endpoint definition for statin was a minimum of three medicine purchases. After searching the website of the Integrative Epidemiology Unit (IEU) OpenGWAS project, Medical Research Council, University of Bristol (https://gwas.mrcieu.ac.uk/) with the keyword “gut microbiota,” a total of 221 species of gut microbiota abundance GWAS data were included in this study. Details of GWAS datasets included in this study are listed in Table 1.

**TABLE 1:** Details of the GWAS included in this study.

Phenotype	Sample size	SNP	Year	Population	GWAS ID
Statin medication	377,277		2023	European	FinnGen
Parabacteroides	7,738	5,564,061	2022	European	ebi-a-GCST90027770
*Ruminococcaceae* UCG-009	14,306	5,418,432	2021	European	ebi-a-GCST90017057
*Coprococcus* 1	14,306	5,649,452	2021	European	ebi-a-GCST90016983
*Desulfovibrio*	7,738	4,925,569	2022	European	ebi-a-GCST90027815
*Ruminococcaceae* UCG-010	14,306	5,545,122	2021	European	ebi-a-GCST90017058
*Veillonellaceae*	14,306	5,688,519	2021	European	ebi-a-GCST90016956

The six species of gut microbiota in the results of this MR study were derived from two studies (PMID: 35115690 and PMID: 33462485). The study (PMID: 35115690) performed a genome-wide association analysis of 412 traits (205 functional pathways and 207 microbial taxa) using 7,738 European samples. They transformed abundances using natural logarithms and regressed them in linear mixed models using SAIGE v.0.38, incorporating age, sex, and the matrix of genetic relationships between participants as covariates. They used SAIGE’s standard setting of back-rank normalization of traits prior to association analyses. Genetic relationship matrices were built with SAIGE using a set of 54,565 SNPs selected from quality control SNPs that were directly genotyped and screened for allele frequency and redundancy (MAF ≥ 0.05, r2 < 0.2, and sliding window = 500 kb).

Microbiome GWAS analyses in another study (PMID: 33462485) were performed in three ways: first, a Spearman correlation between the SNP dose and alpha diversity metrics was utilized in their GWAS of three microbiome alpha diversity metrics (Shannon, Simpson, and inverse Simpson), adjusted for age, sex, technological covariates, and genetic principal components (PCs). Second, a Spearman correlation was used to identify the loci affecting the covariate-adjusted abundance of bacterial taxa, excluding samples with zero abundance (mbQTLs). Third, they identified loci associated with the probability that a bacterial taxon might be present (mbBTLs). For the mbBTL analysis, they used a robust methodology for binary trait GWAS: a two-stage approach consisting of fast correlation screening and logistic regression analysis.

### 2.2 Selection of instrumental variables

We identified eligible SNPs highly associated with statins as instrumental variables (IVs) according to the following three rules: 1) TwoSampleMR, an R software package, was used to select SNPs that were significantly associated with 221 gut microbiota, which were set to a *p*-value of less than 5 × 10^−8^ for highly associated candidate-eligible IVs. 2) We clumped SNPs that were highly correlated with statins in order to ensure that the assignment of different SNPs was completely randomized. In SNPs, within a distance of 1 MB, the parameter for clump processing was set to [LD] r^2^ < 0.001. 3) To avoid weak instrument bias in candidate SNPs, we used the *F*-statistic (*F* = R^2^ × (N–2)/(1–R^2^)) to assess weak instrumental variable effects and excluded SNPs with *F* < 10.

Furthermore, we retrieved these SNPs highly associated with statins and excluded 68 SNPs associated with cholesterol, HDL, LDL, lipid metabolism, coronary heart disease, and metabolic syndrome, respectively, according to the PhenoScanner database (http://www.phenoscanner.medschl.cam.ac.uk/).

### 2.3 Mendelian randomization analysis

In this study, we used three Mendelian randomization analysis methods [inverse variance weighting (IVW), MR-Egger, and weighted median] to assess the potential causal effects of statins on gut microbiota. IVW, which estimates the causality of exposure on the outcome by comparing the Wald ratio of each SNP, is the most commonly used method for MR analysis evaluation, and therefore, it is designated as the primary MR analysis method. Although the results of MR-Egger and weighted median could be a valid complement to IVW, the results of IVW had the highest priority. If the results of our MR analysis were inconsistent, a tightened instrument *p*-value (*p* < 1 × 10^−9^) threshold would be adopted.

### 2.4 Sensitivity analysis

The heterogeneity test ensured that Mendelian randomization results were not influenced by the heterogeneity of the sample (Cochran’s Q statistic and *p*-value > 0.05), and the MR-Egger intercept test (*p*-value > 0.05) was used to estimate horizontal pleiotropy. After that, MR Pleiotropy RESidual Sum and Outlier (MR‐PRESSO) was employed to detect and exclude outlier SNPs and estimate the corrected causal effects. The funnel plot was used to evaluate the symmetry from a visual scatter plot. A leave-one-out analysis was conducted to determine whether any individual SNP had a significant independent influence on the validity of MR results.

### 2.5 Statistical analysis

The robustness and reliability of the methodology of the study were checked and found to be consistent with the STROBE-MR checklist (Skrivankova et al., 2021). This study satisfies the three main assumptions of Mendelian randomization: 1) the genetic variant must reliably associate with the risk factor; 2) the genetic variant must not be associated with any known or unknown confounders; and 3) the genetic variant must influence the outcome only through the risk factor and not through any direct causal pathway.

All analyses for this study were conducted using TwoSampleMR (version 0.5.7) in R software (version 4.2.3). *p* < 0.05 is considered nominally significant.

## 3 Results

### 3.1 Genetic IVs enrolled in this study

**FIGURE 1:**
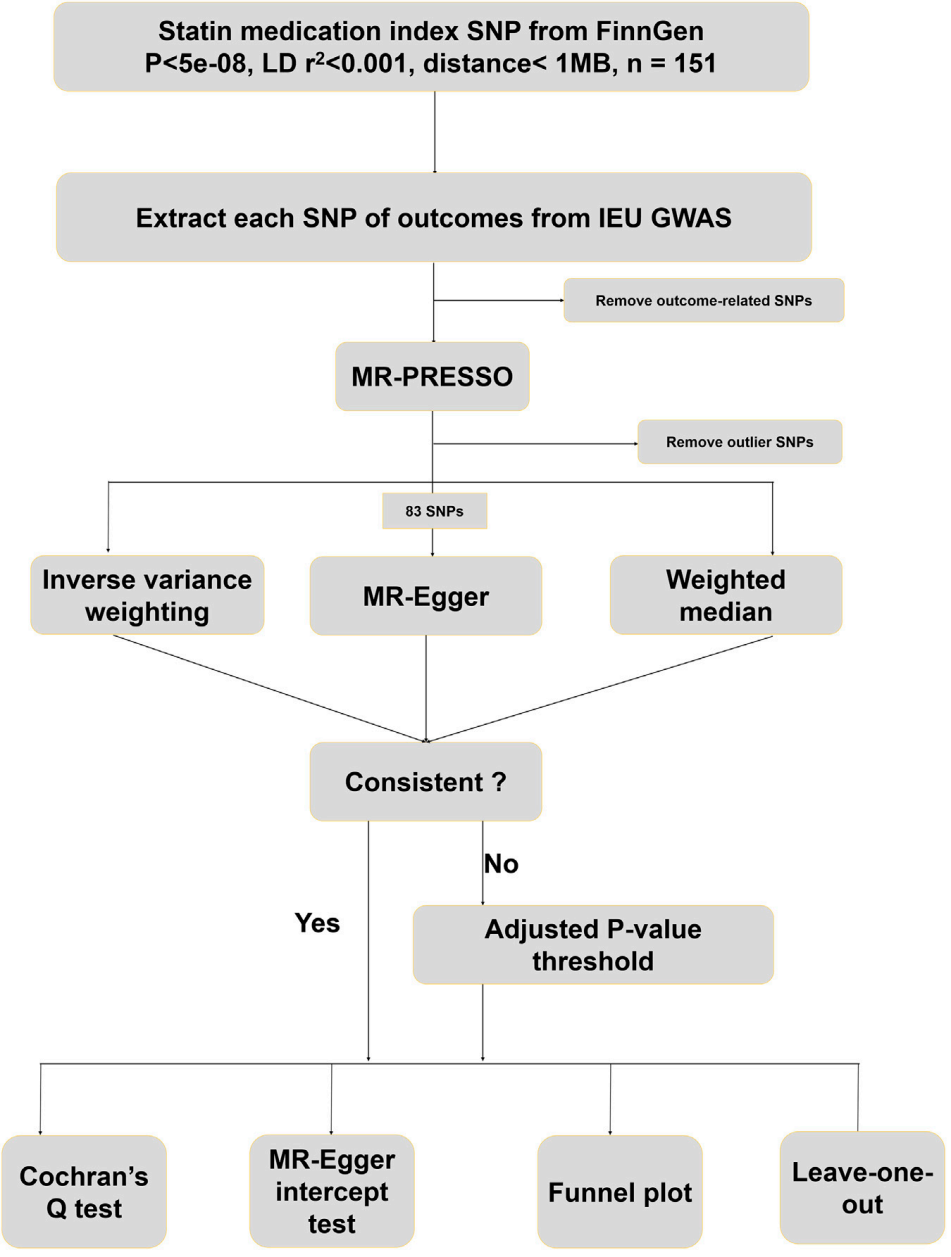
Design and flowchart of this study.

The design protocol for this study is shown in Figure 1. A total of 127,169 participants were included in the FinnGen GWAS database for statin medication. At first, 151 SNPs highly associated with statin medication were screened based on a significance threshold (*p* < 5 × 10^−8^), LD test (r^2^ < 0.001 and physical distance < 1 MB), and F-statistics (>10). The predominant mechanism of statin effects in the body is the reduction of cholesterol levels by inhibiting HMG-CoA reductase enzyme activity, a key enzyme in the cholesterol synthesis pathway. We searched for all SNP-associated phenotypes on the PhenoScanner website in order to remove instrumental variables associated with cholesterol, HDL, LDL, lipid metabolism, coronary heart disease, and metabolic syndrome. Eventually, 68 SNPs were excluded from this study, and the phenotypes associated with the remaining 83 SNPs included in the study are shown in Additional file 1: Supplementary Table S1. These retained 83 SNPs were then harmonized with 221 species of gut microbiota abundance GWAS data extracted from the IEU database to exclude palindrome structure. Finally, none outliers were detected in the MR-PRESSO outlier test.

### 3.2 Causal effects of statin medication on gut microbiota abundance

**FIGURE 2:**
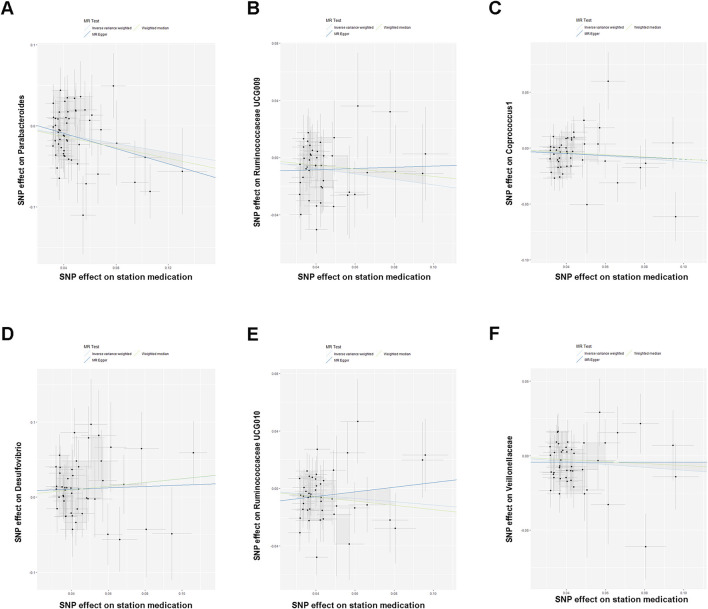
**(A–F)** Scatterplot showing the results of the MR analysis on the association between statin medication and gut microbiota abundance.

We conducted Mendelian randomization analysis (IVW, MR-Egger, and weighted median) on these 83 statin medication-associated SNPs using 221 species of gut microbiota abundance GWAS data from IEU. We found that genetic susceptibility to long-term statin medication intake was causally related to gut microbiota abundance (Figure 2).

The IVW results indicated that statin medication was negatively correlated with five species of gut microbiota abundance: *Parabacteroides* (Beta_IVW_ = −0.2745, 95% CI = (−0.4422,−0.1068), and *P*_IVW_ = 0.0013), *Ruminococcaceae UCG-009* (Beta_IVW_ = −0.1904, 95% CI = (−0.3255, −0.0553), and *P*_IVW_ = 0.0057), *Coprococcus 1* (Beta_IVW_ = −0.1212, 95% CI = (−0.2194, −0.0231), and *P*_IVW_ = 0.0154), *Ruminococcaceae UCG-010* (Beta_IVW_ = −0.1149, 95% CI = (−0.2238, −0.0060), and *P*_IVW_ = 0.0385), and *Veillonellaceae* (Beta_IVW_ = −0.0970, 95% CI = (−0.2238, 0.0060), and *P*_IVW_ = 0.0400) and positively correlated with one species of gut microbiota: *Desulfovibrio* (Beta_IVW_ = 0.2452, 95% CI = (0.0299, 0.4606), and *P*_IVW_ = 0.0255) (Table 2).

**TABLE 2:** Results of Mendelian randomization analysis of statin medication on gut microbiota abundance.

Gut microbiota	nSNP	*p*-value	Beta	Se	Beta 95% IC	Pleiotropy *p*-value	MR-PRESSO *p*-value
Parabacteroides						0.4479	0.4145
IVW	54	0.0013	−0.2745	−0.0594	(−0.4422, −0.1068)		
MR-Egger	54	0.0868	−0.4694	−0.1012	(−0.9965, 0.0577)		
Weighted median	54	0.0083	−0.3349	−0.1021	(−0.5839, −0.0859)		
*Ruminococcaceae* UCG-009						0.3730	0.7885
IVW	44	0.0057	−0.1904	−0.0919	(−0.3255, −0.0553)		
MR-Egger	44	0.8753	0.0421	−0.1454	(−0.4819, 0.5662)		
Weighted median	44	0.1676	−0.1323	−0.1129	(−0.3202, 0.0556)		
*Coprococcus* 1						0.8426	0.1928
IVW	45	0.0154	−0.1212	−0.0491	(−0.2194, −0.0231)		
MR-Egger	45	0.6736	−0.0833	−0.0571	(−0.4686, 0.3019)		
Weighted median	45	0.1465	−0.0987	−0.0585	(−0.2320, 0.0345)		
*Desulfovibrio*						0.6778	0.5888
IVW	48	0.0255	0.2452	−0.0819	(0.0299, 0.4606)		
MR-Egger	48	0.8151	0.0907	−0.0906	(−0.6652, 0.8467)		
Weighted median	48	0.1304	0.2491	−0.0543	(-0.0073, 0.5720)		
*Ruminococcaceae* UCG010						0.1888	0.2823
IVW	45	0.0385	−0.1149	−0.0559	(−0.2238, −0.0060)		
MR-Egger	45	0.4552	0.1614	−0.0216	(−0.2584, 0.5812)		
Weighted median	45	0.0606	−0.1464	−0.0270	(−0.2995, 0.0065)		
Veillonellaceae						0.5889	0.4778
IVW	45	0.0400	−0.0970	−0.0560	(−0.2238, 0.0060)		
MR-Egger	45	0.9994	−0.0001	0.0048	(−0.2584, 0.5812)		
Weighted median	45	0.3321	−0.0658	−0.0612	(−0.2995, 0.0065)		

In addition, the results of the MR-Egger analysis showed that the findings of *Parabacteroides* (Beta_Weighted median_ = −0.3349, 95% CI = (−0.5839, −0.0859), and *P*_Weighted median_ = 0.0255) in the weighted median analysis were also consistent with those of IVW (Table 2). Meanwhile, we observed a direction inconsistency between MR-Egger and IVW in the results for *Ruminococcaceae UCG-009* and *Ruminococcaceae UCG-010*. In addition, when we applied a stricter *p*-value (*p* < 1 × 10^−9^), this trend of inconsistency decreased significantly. More detailed results of IVW, MR-Egger, and weighted median analyses are shown in Table 2.

The six species of gut microbiota identified in the results of this MR study were derived from PMID: 35115690 and PMID: 33462485. The raw data were filtered, and uploaded only the GWAS for 14,306 Europeans (out of 18,340 mixed populations) were uploaded to the IEU database, resulting in 221 GWAS related to the gut microbiota. Considering that the GWAS data for statins were derived from the FinnGen database, the population in this two-sample Mendelian randomization study originated from the same ethnic group, with minimal differences between the populations.

### 3.3 Sensitivity analysis

**FIGURE 3:**
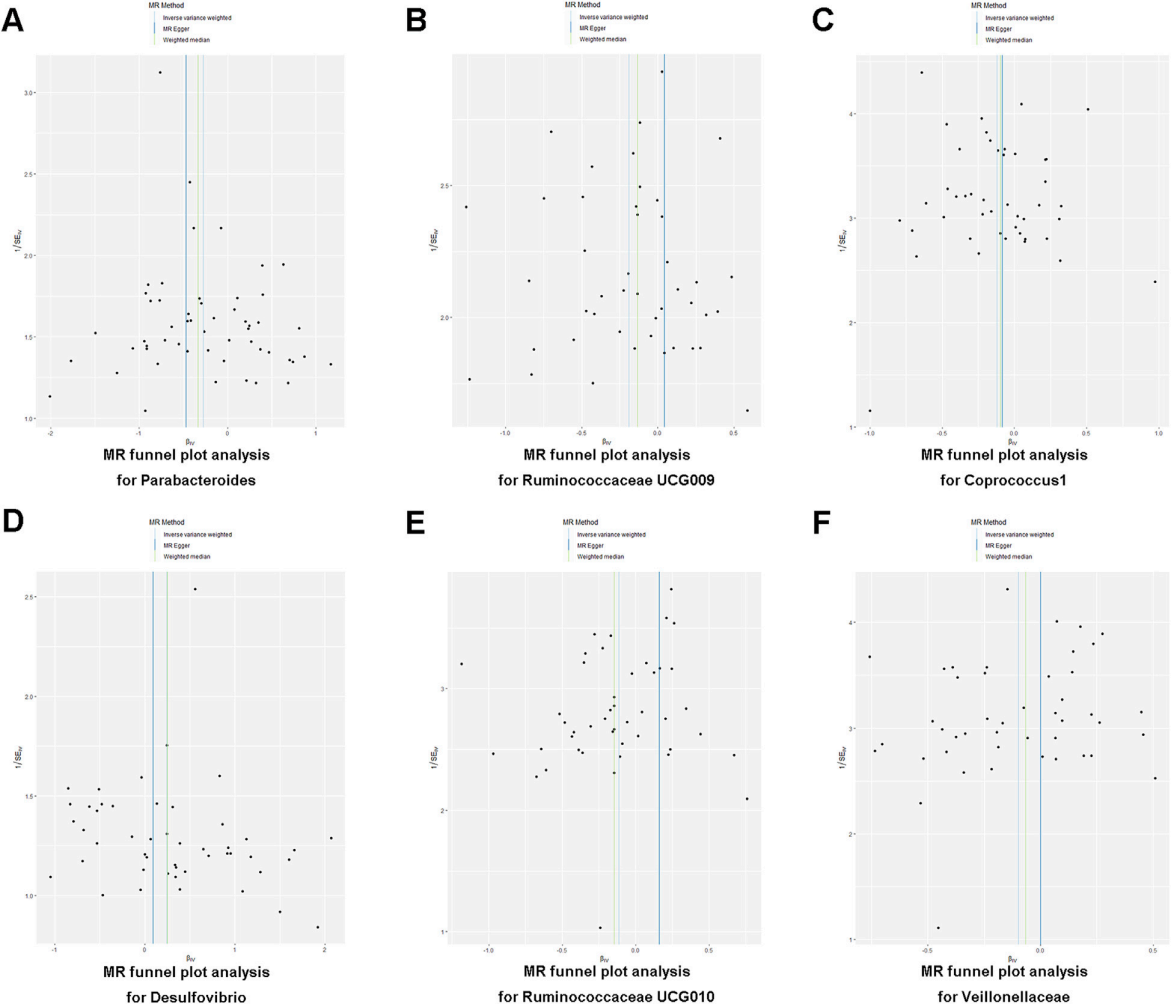
**(A–F)** Funnel plot illustrating the association between statin medication and gut microbiota abundance.

**FIGURE 4:**
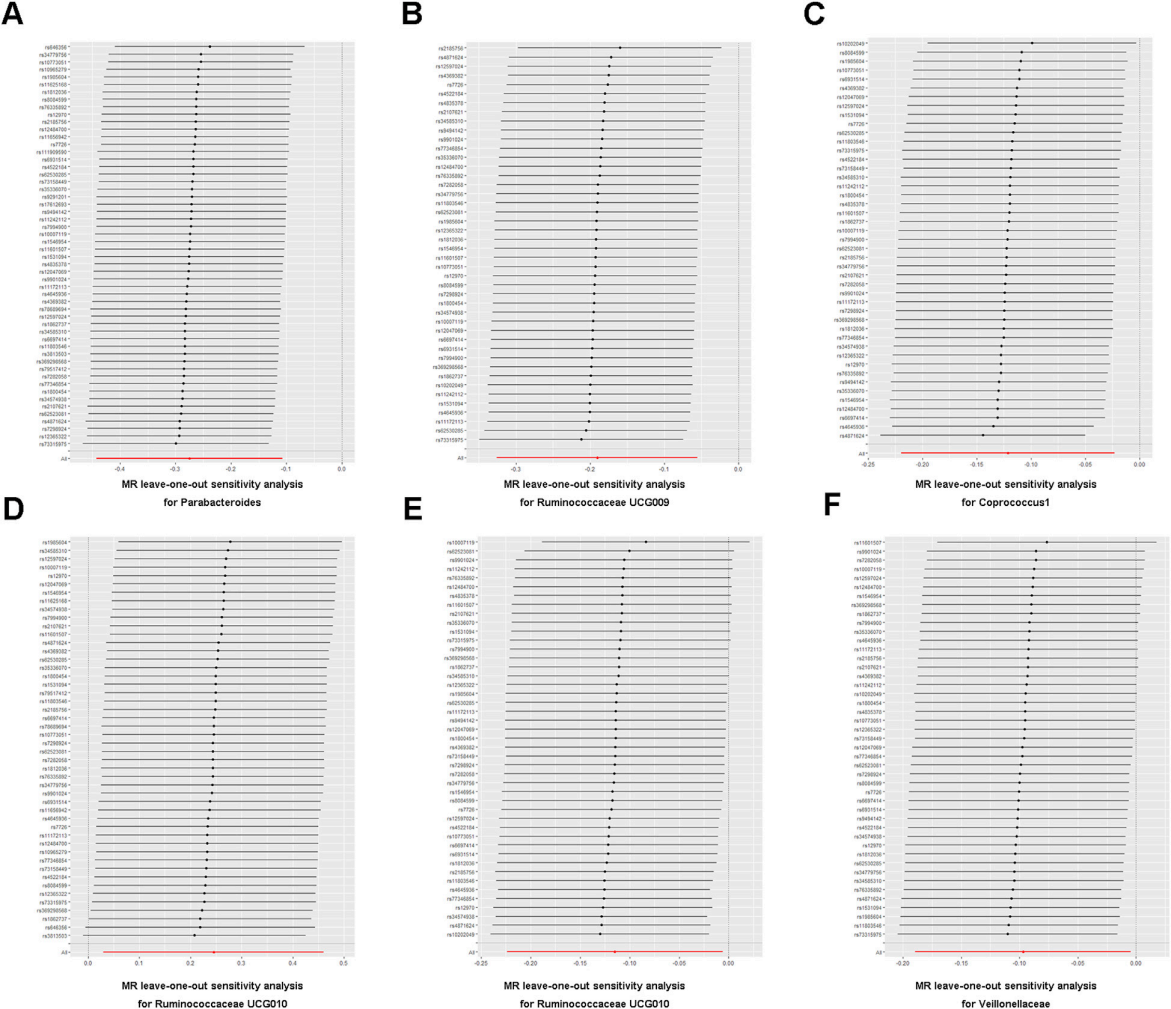
**(A–F)** Leave-one-out analysis of the association between statin medication and gut microbiota abundance.

To determine the credibility of our causal inferences, we conducted a series of heterogeneity and pleiotropy analyses. The MR-PRESSO global and outlier tests did not find potential horizontal pleiotropy and statistically significant outliers (Table 3). The Cochran’Q statistic was used to analyze the heterogeneity of this study, and there were no statistically significant findings (*p* > 0.05) (Table 3). The results of the MR-Egger intercept indicated that there was no significant horizontal pleiotropy in this MR study (Egger intercept < 0.01 and *p* > 0.05) (Table 3). As shown in the funnel plot, the scatter plot of effect sizes for each SNP was roughly symmetric (Figure 3). The “leave-one-out” plot calculated the meta-effects of the remaining SNPs by progressively eliminating each SNP and found that no single instrumental variable was able to drive a causal effect between statin medication and gut microbiota abundance (Figure 4). In conclusion, the results of both the heterogeneity and pleiotropy analyses mentioned above validated the credibility of this MR analysis.

**TABLE 3:** Results of heterogeneity and pleiotropy analysis of causal effects of statin medication on gut microbiota abundance.

Gut microbiota	MR-Egger_ Intercept	Egger_intercept_pval	IVW_Cochrane_Q	IVW_Cochrane_Q_pval	MR-PRESSO *p*-value
*Parabacteroides*	0.0093	0.4479	54.7099	0.4094	0.4145
*Ruminococcaceae* UCG-009	−0.0101	0.3730	35.6694	0.7785	0.7885
*Coprococcus* 1	−0.0016	0.8426	51.7230	0.1699	0.1928
*Desulfovibrio*	0.0070	0.6778	44.3841	0.5815	0.5888
*Ruminococcaceae* UCG-010	−0.0120	0.1888	48.9264	0.2818	0.2823
*Veillonellaceae*	−0.0042	0.5889	43.8242	0.479	0.4778

## 4 Discussion

Increasingly, research attention has shifted to the study of the gut microbiota. Growing evidence suggests that dysbiosis of the gut microbiota is associated with a wide range of diseases, including cancer, autoimmune disorders, and cardiovascular diseases. Gut microbiota has been shown to exhibit competence in disease processes via a variety of approaches and pathways. First, the gut microbiota is recognized as a metabolic organ that regulates the state of the whole organism by influencing the metabolism and energy absorption in the gut. It plays a pivotal role by housing a plethora of essential digestive enzymes that are capable of metabolizing various substrates, such as dietary fiber, proteins, and amino acids, thereby sustaining the host’s energy homeostasis (Adak and Khan, 2019). Second, the gut microbiota modulates the immune homeostasis of the gut by regulating the integrity of the intestinal barrier and the secretory function of immune cells. The gut microbiota engages in interactions with immune cells and epithelial cells, increasing specific immune responses (Takiishi et al., 2017). This interaction leads to a harmonious balance between tolerance and effector immune functions. Third, the composition and diversity of the gut microbiota significantly influence the function of enteroendocrine cells (EECs) in regulating the production and secretion of gut hormones such as cholecystokinin (CCK) and glucagon-like peptide-1 (GLP-1). In addition, gut microbiota has also been reported to be able to damage the structure of DNA and modulate the expression levels of specific genes to influence the course of disease. Numerous studies have demonstrated the pivotal role of gut microbiota dysregulation and its associated metabolite profile in the development of various diseases, such as inflammatory bowel diseases, neurodegenerative disorders, and cervical cancer (Zhou et al., 2022). In addition to the intestinal flora, minor components of the gut microbiota (archaea, virome, and mycobiota) have also been implicated in statins. For example, lovastatin could influence the cell membrane biosynthesis of methanogens by producing β-hydroxy acid lovastatin with the assistance of intestinal anaerobes to inhibit methane production and methanogen growth (Demonfort et al., 2017).

Although traditional biomarkers have been widely used for disease diagnosis and treatment, there are still issues regarding their insufficient sensitivity, lack of specificity, and limited predictive ability. The clinical field continues to seek novel biomarkers that are minimally invasive, cost-effective, easily accessible, and better suited for early disease diagnosis and targeted therapy. An ideal clinical biomarker should be rapid, quantitative, objective, reproducible, non-invasive, and highly accurate in predicting disease states across various populations or ethnicities (Califf, 2018). The advancement of metagenomic sequencing and metabolomics technology has significantly deepened our understanding of host–microbiota interactions within the human gut. It is increasingly recognized that the gut symbiosis microbiota plays a vital role in the fundamental physiological functions of the human body. Recent studies have unveiled alterations in the composition and abundance of the gut microbiota in colorectal cancer (CRC) patients, whereas animal model experiments have further confirmed the roles of several bacteria in the development and progression of colorectal carcinogenesis, including *Fusobacterium nucleatum*, as well as specific strains of *Escherichia coli and Bacteroides fragilis* (Castellarin et al., 2012; Wu et al., 2009; Arthur et al., 2012). Ren *et al* introduced a metagenomic analysis of fecal microbiota in hepatocellular carcinoma (HCC). They identified optimal microbial markers and developed an HCC classifier. The validity of this classifier was substantiated through rigorous testing on HCC samples, exhibiting diverse degrees of progression, as well as on cross-regional samples (Ren et al., 2019). These findings offer novel insights into the clinical utilization of the gut microbiota as potential biomarkers for disease screening, prognosis, or prediction. Therefore, diagnostic models based on the biomarkers of microbiota have great potential in clinical practice and provide new approaches for early prevention and diagnosis, which has a favorable prospect in clinical application.

In this study, we used multiple two-sample Mendelian randomization analyses to provide novel evidence for validating statin medication and diverse gut microbiota abundance. Mendelian randomization is the method of cause-and-effect inference based on the instrumental variable analysis of genetic variation, and its fundamental theory is that the random assignment of genotypes according to Mendel’s laws of inheritance can be used to infer the influence of susceptibility factors on diseases and phenotypes (Birney, 2022; Wootton et al., 2022; Mukamal et al., 2020). We screened SNPs highly correlated with both statin medication and gut microbiota abundance as instrumental variables to assess the causal relationship between them. In this study, we innovatively selected and, for the first time, enrolled a statin medication GWAS dataset of 377,277 participants from the FinnGen research project and 221 species of gut microbiota abundance GWAS data from the IEU database. This makes the study a summary of a very large population sample. In addition, the advantages of this MR study include using genetic variation as an instrumental variable, which reduces measurement errors with its effect; clarifying the direction of statin medication causal effect on gut microbiota abundance; removing confounding factors such as smoking, alcohol consumption and cancer; and employing FinnGen and IEU GWAS data without the need for human interventions in large populations and ethical review (Tin and Köttgen, 2021).

As we mentioned in the Methods section, this study adopted a rigorous selection scheme to obtain SNPs that were highly associated with statin. Furthermore, considering the mechanism and application population of statin medication, we checked all SNP traits in the PhenoScanner database and excluded all the SNPs related to cholesterol, HDL, LDL, lipid metabolism, coronary heart disease, and metabolic syndrome. After that, we harmonized SNPs associated with exposure to gut microbiota abundance GWAS from IEU and removed the palindrome sequence. These abovementioned steps were necessary and effective because they ensured a high correlation between SNPs and exposure, as well as eliminating the bias of confounders. Then, we conducted a two-sample MR analysis, and the IVW MR analysis results showed that statin therapy may cause the downregulation of *Parabacteroides, Ruminococcaceae UCG-009*, *Coprococcus 1*, *Ruminococcaceae UCG-010*, and *Veillonellaceae* and upregulation of *Desulfovibrio* abundance. Among them, the weighted median result for *Parabacteroides* was consistent with its IVW results. Meanwhile, Cochran’s Q test, MR-Egger intercept test, leave-one-out analysis, funnel plot, and MR-PRESSO were also conducted to detect heterogeneity or pleiotropy, and no significant finding was detected.

After retrieving these gut microbiotas in the Disbiome database (Janssens et al., 2018), a database covering microbial composition changes in different types of diseases based on PubMed, we recognized a 16S-rRNA-sequencing correlation between the dysregulation of these flora and the development of many diseases. For example, *Parabacteroides* are a group of anaerobic Gram-negative bacilli that can influence the development and function of the host immune system and participate in the regulation of host immunomodulation (Wei et al., 2023; Lei et al., 2021). *Parabacteroides* can also inhibit the growth of potentially pathogenic microorganisms in the intestinal tract by competing for resources and ecological niches, and this competitive exclusion contributes to the maintenance of intestinal microecological balance (Peterson et al., 2020). *Ruminococcaceae* are a family of Gram-positive bacteria that play a pivotal role in the disintegration of cellulose, the production of short-chain fatty acids (e.g., propionic, acetic, and butyric acids), and other organics that can assist the host in digesting cellulose and other complex carbohydrates that are difficult to degrade in the plant-based diet (Pan et al., 2021). In addition, *Ruminococcaceae* produce such metabolites as short-chain fatty acids that have a positive impact on the host’s intestinal health and immune system (Sulit et al., 2023), and its downregulation in abundance was seen in the 16S RNA sequencing in multiple sclerosis, chronic fatigue syndrome, and cirrhosis. Other disorders associated with gut microbiota can be obtained by searching the Disbiome database (Janssens et al., 2018).

Our MR results revealed a causal relationship between statin medication and gut microbiota abundance, which is closely involved in the development of many diseases. This study provides a new strategy for predicting and minimizing the occurrence of certain diseases by regularly sequencing the gut microbiota in patients taking long-term statin medication because the population base of long-term statin drug users is so large that such testing is necessary and effective. However, there are some limitations to our study. The populations included in this study were European, and the results may not be generalizable. Our study explored the causal relationship using genetic variation as an instrumental variable, and a randomized controlled study of scale is still required to confirm its plausibility. In addition, the specific molecular mechanisms of statin regulation of gut microbiota remain to be investigated.

## 5 Conclusion

This study, for the first time, demonstrated a causal relationship between statin medication and gut microbiota through a Mendelian randomization analysis. This analysis revealed that statin therapy modulates the downregulation of five gut microbiota species and the upregulation of one. Clinicians are advised to predict and guard against certain complications by periodically sequencing gut microbiota in patients undergoing long-term statin therapy.

## Data Availability

The original contributions presented in the study are included in the article/Supplementary Material; further inquiries can be directed to the corresponding authors.

## Abbreviations

MR, Mendelian randomization; GWASs, genome-wide association studies; IEU, Integrative Epidemiology Unit; IVW, inverse variance weighting; CI, confidence interval; SNP, single-nucleotide polymorphism; IV, instrumental variable; HDL, high-density lipoprotein; LDL, low-density lipoprotein; CCK, cholecystokinin; GLP-1, glucagon-like peptide-1; CRC, colorectal cancer; HCC, hepatocellular carcinoma.
